# The influence of genetic factors on education health and care plan obtainment for pupils with intellectual developmental disabilities

**DOI:** 10.1038/s41598-026-36227-5

**Published:** 2026-02-15

**Authors:** Irene O. Lee, Jeanne Wolstencroft, Harriet Housby, Marianne B. M. van den Bree, Samuel J. R. A. Chawner, Jeremy Hall, Michael J. Owen, IMAGINE ID Consortium, Irene O. Lee, Jeanne Wolstencroft, Marianne B. M. van den Bree, Samuel J. R. A. Chawner, Jeremy Hall, Michael J. Owen, Josh Hope-Bell, F. Lucy Raymond, David H. Skuse, David H. Skuse

**Affiliations:** 1 Paediatric Mental Health Sciences Centre, Great Ormond Street Institute of Child Health, University College London, London, UK; 2 The Division of Psychology and Language Sciences, Faculty of Brain Sciences, University College London, London, UK; 3 Centre for Neuropsychiatric Genetics and Genomics, Division of Psychological Medicine and Clinical Neurosciences, Cardiff University, Cardiff, UK; 4 Neuroscience and Mental Health Innovation Institute, Division of Psychological Medicine and Clinical Neurosciences, Cardiff University, Cardiff, UK; 5 IMAGINE ID Consortium, GB, UK; 6 Department of Medical Genetics, University of Cambridge, Cambridge, UK

**Keywords:** Intellectual developmental disabilities, Genetic inheritance, Socioeconomic status, Education health and care plan, Inequality, Parental education level, Human behaviour, Genetics research, Paediatric research, Medical research, Health care, Diagnosis, Health care economics, Health services, Paediatrics, Patient education

## Abstract

The study aimed to investigate the hypothesis that the Education, Health and Care Plan (EHCP) obtainment for pupils with intellectual developmental disabilities (IDD) of genetic aetiology was influenced by genetic factors and their family’s socioeconomic status. We recruited 2738 participants with IDD of genetic aetiology aged between 6–28 years old (mean age 14 years, 56% male). The data used included genetic inheritance information, primary special educational needs type, the age when the EHCP was granted, parental education level, free school meal eligibility and a family index of multiple deprivation score. Only 78% of participants were eventually granted EHCPs. Those with an inherited variant waited significantly longer and were less likely to receive an EHCP than those with a *de novo* variant. They were significantly more likely to be living in more deprived areas, and their parents had attained lower levels of education compared with those whose IDD was caused by an equivalent *de novo* genetic anomaly. This unique cohort study demonstrated that the obtainment of an EHCP in families seeking additional educational support for children with IDD of genetic origin is inequitable and is influenced by social and genetic factors.

**Supplementary Information:**

The online version contains supplementary material available at 10.1038/s41598-026-36227-5.

## Introduction

Intellectual developmental disability (IDD) is characterised by significantly impaired intellectual functioning and deficits in adaptive behaviours^1–3^. There is no identifiable aetiology in up to 60% of cases^4^, but 25 to 50% are attributable to genetic factors; and this proportion is rising with recent advances in genetic technologies^2,4–6^. The types of genetic aetiology include a copy number variation (CNV) or a single nucleotide variant (SNV)^1,7,8^. CNVs were previously detected by comparative genomic hybridization array and small structural changes, which are not necessarily pathogenic, are duplications and deletions of small DNA segments^1^. A couple of decades ago, the introduction of exome sequencing first enabled the detection of SNV for associated pathology where the function of a critical gene is disrupted^7,9^. Identification of mutations in protein coding regions has since revealed a wide variety of genes that underlie neurodevelopmental disorders^9–11^. Subsequent studies have found that up to 85% of pathological single nucleotide mutations result in IDD^1^. In the UK, the National Health Service (NHS) clinical genetics services have recently introduced a diagnostic service using whole genome sequencing for children with complex developmental presentations^2,12^, allowing a more in-depth examination of SNVs, CNVs, indels and structural variants^1^. Genetic testing of family members including a trio analysis, i.e. parents are tested at the same time as their child^12^, reveal insights into the genetic inheritance of the variant. If a genetic change is identified in the child but not in either parent this is termed “*de novo*”. Both *de novo* and inherited SNV and CNV mutations have been associated with IDD^1,13,14^.

Children and young people (CYP) with IDD are impacted in all key areas of life, including health, education and wellbeing^15^. They have greater difficulties both in their daily life skills and emotional or behavioural adjustment than the general population^16,17^. There is a higher prevalence of IDD in areas of lower socioeconomic status (SES)^4,17^, influenced by environmental factors such as maternal education level, access to education/opportunity and access to healthcare^4,18,19^. Children with IDD usually need additional educational support; in the UK those identified with special educational needs (SEN) are eligible for an Education, Health and Care Plan (EHCP). Nevertheless, EHCPs are not provided to all pupils with substantial additional educational needs^20^ and recent research has shown regional and socioeconomic inequality in EHCP provision^16^. Many children do not receive an EHCP after the first application. If the pupil’s parents, carers or the young person themselves disagree with the local authority’s decision, they can appeal to the Special Educational Needs and Disability Tribunal^21^. Waiting times between the point at which SEN are identified and an EHCP is granted are highly variable^22,23^. Delays or lack of provision of this additional educational support can lead to detrimental outcome in education, health, and developmental progress^24–26^.

In this unique nationwide study, we obtained data from Regional Genetics Centres in England concerning children who had been identified as having IDD that were caused by pathogenic SNV or CNV, over the period 2015–2020. We linked their genetic anomalies to key educational indicators from the Department for Education’s National Pupil Database (NPD), and to information about parental educational background and family neighbourhood. We had three main objectives: (1) to correlate participating children’s daily living skills (measured by Adaptive Behaviour Assessment System), emotional and behavioural adjustment (evaluated by Strength and Difficulties questionnaire) and severity/types of learning difficulties (specified by the child’s primary SEN type) with genetic factors (their variant type and whether it was inherited or *de novo*); (2) to investigate whether the *de novo* or inherited status of their condition influenced whether (and how quickly) they were granted an EHCP irrespective of the variant type; (3) to assess whether there was an association between disparities in EHCP obtainment and individual and/or family factors. We looked at the potential influence of severity/types of learning difficulties and the family’s socioeconomic status (indicated by index of multiple deprivation, free school meal eligibility and parental education level). We hypothesised that both genetic and environmental factors such as socioeconomic status could affect whether and when an EHCP would be granted.

## Results

### Age at genetic diagnosis

The mean age at genetic diagnosis in this cohort was 6.8 (4.3) years [mean (SD); 95% CI = 6.6–6.9] with no difference between the sexes, *t*(2061) = -0.54, *p* = 0.589, *d* = 4.314. The average age at genetic diagnosis was substantially later for those with an SNV [9.0 (4.7) years] compared to those with a CNV [5.8 (3.9) years], *t*(2737) = 14.68, *p* < 0.001, *d* = 4.2. School records showed that participants with an SNV were nevertheless identified as having special educational needs at a similar age [5.5 (1.7) years] to those with a CNV [5.7 (1.7) years], *t*(2697) = −3.028, *p* = 0.003, *d* = 1.72. Participants with a familial variant were diagnosed with a genetic disorder at a significantly younger age [6.4 (4.1) years] than those with a *de novo* variant [7.1 (4.8) years], *t*(2737) = 3.041, *p* = 0.001, *d* = 4.574. Those with a familial variant were identified with SEN at a mean age of 5.9 (1.9) years, slightly later than those with *de novo* variants [5.5 (1.6) years], *t*(2697) = -4.699, *p* < 0.001, *d* = 1.714.

The mean age at genetic diagnosis of those with a familial CNV was 5.5 (4.1) years, a significantly younger age than those with a *de novo* CNV [6.3 (3.8) years], familial SNV [7.9 (5.5) years] and *de novo* SNV [9.3 (4.6) years], *F*(2096) = 86.55, *p* < 0.001. Those with a familial CNV were identified with SEN at a mean age of 5.5 (1.6) years, slightly earlier than those with *de novo* CNV [5.9 (1.9) years], *t*(1514) = -3.785, *p* < 0.001, *d* = 1.74; whereas pupils with a familial SNV were identified with SEN at a mean age of 5.9 (1.8) years, significantly older than those with *de novo* SNV [5.0 (1.4) years], *t*(271) = 3.132, *p* = 0.003, *d* = 1.49.

### Genetic factors and daily life skills – Adaptive Behaviour Assessment System

The General Adaptive Composite (GAC) scores to assess the daily life skills were obtained from the Adaptive Behaviour Assessment System, version 3 (ABAS-3). The mean GAC standard scores of all the groups were in the ‘extremely low’ range, which encompasses just 2.2% of children from the general population^27^, [mean (SD)] [SNV: 60.2 (14), CNV: 64.8 (13); *de novo* CNV: 64.1 (14), familial CNV: 65.0 (13); see Table 1]. This implies participants in our study usually had very poor adaptive skills relative to the general population of children at a similar age. Proportionately more of those with an SNV had scores within the ‘extremely low’ range (SNV = 75.2% vs CNV = 66.4%), as did those with a *de novo* CNV (76.6%) [*de novo* (72.1%) vs familial (64.3%)].

**Table 1:** Comparison of the General Adaptive Composite (GAC) standard scores of the participants with different variant types and genetic inheritance and inheritance variant groups.

			GAC Standard Score Group*				
			Extremely Low (< 70)	Low (71–80)	Below Average (81–90)	Average (91–110)	Mean (SD) [GAC Standard Score Group]
**Variant type**	**SNV**	Count	272	50	24	15	359
		% within group	75.20%	13.90%	6.70%	4.20%	60.2 (14) [Extremely Low]
	**CNV**	Count	546	171	75	30	836
		% within group	66.40%	20.80%	9.10%	3.60%	64.8 (13) [Extremely Low]
**Genetic inheritance**	***De novo***	Count	513	115	58	26	712
		% within group	72.10%	16.20%	8.10%	3.70%	62.2 (14) [Extremely Low]
	**Familial**	Count	218	81	27	13	339
		% within group	64.30%	23.90%	8.00%	3.80%	64.6 (13) [Extremely Low]
**Inheritance variant groups**							
**Familial SNV**		Count	28	7	3	3	41
		% within group	68.30%	17.10%	7.30%	7.30%	62.2 (14) [Extremely Low]
***De novo*** ** SNV**		Count	233	42	18	11	304
		% within group	76.60%	13.80%	5.90%	3.60%	59.7 (14) [Extremely Low]
**Familial CNV**		Count	190	74	24	10	298
		% within group	63.80%	24.80%	8.10%	3.40%	65.0 (13) [Extremely Low]
***De novo* CNV**		Count	280	73	40	15	408
		% within group	68.60%	17.90%	9.80%	3.70%	64.1 (14) [Extremely Low]

*General Adaptive Composite (GAC) standard scores from ABAS-3 were categorised descriptively into different standard score groups to indicate an individual’s adaptive abilities^27^.

### Genetic factors and emotional behavioural difficulties – Strengths and Difficulties Questionnaire (SDQ)

Table 2 shows the comparisons of the total and subscales of the SDQ scores in different groups of variant types and inheritance. These have been categorised into bands indicating degrees of severity, based on data from UK population norms^28,29^ (see categorisation bands in Supplementary Note S1). For those with familial CNV (see Table 2), their total difficulties scores were on average in the ‘very high’ range; their emotional symptoms, conduct problems, hyperactivity and peer problems were in the ‘high’ band category. All SDQ subscale mean scores were significantly greater than the equivalent subscale scores in children with *de novo* CNV (all *p*-values < 0.001, Table 2), indicating that they had more severe difficulties.

**Table 2:** Comparisons of Strengths and Difficulties Questionnaire (SDQ) scores and categorisation bands of participants between SNV and CNV, between *de novo* and familial variants, between inheritance of SNV, and between inheritance of CNV. *UK Norm (80% population)^28,29^.

		SDQ score mean (SD) [band]	N	Total difficulties	Emotional symptoms	Conduct problems	Hyperactivity	Peer problems	Prosocial behaviour
		*UK norm		0–13 [Average]	0–3 [Average]	0–2 [Average]	0–5 [Average]	0–2 [Average]	8–10 [Average]
**Variant type**		**SNV**	637	18.2 (6.1) [High]	3.9 (2.7) [Close to Average]	2.6 (1.9) [Close to Average]	7.5 (2.2) [Slightly Raised]	4.1 (2.2) [High]	4.9 (2.9) [Very Low]
		**CNV**	1730	21.0 (6.8) [Very High]	4.9 (2.8) [Slightly Raised]	3.7 (2.5) [Slightly Raised]	7.8 (2.2) [Slightly Raised]	4.6 (2.3) [High]	5.3 (2.8) [Very Low]
Between variant types		*t*		-9.684	-7.436	-10.947	-3.308	-4.811	-3.333
		*p*-value		< *0.001*	< *0.001*	< *0.001*	< *0.001*	< *0.001*	< *0.001*
**Genetic inheritance**		***De novo***	1216	18.6 (6.5) [High]	4.1 (2.8) [Slightly Raised]	2.9 (2.1) [Close to Average]	7.5 (2.3) [Slightly Raised]	4.2 (2.3) [High]	5.1 (2.9) [Very Low]
		**Familial**	691	22.4 (6.4) [Very High]	5.4 (2.7) [High]	4.1 (2.5) [High]	8.1 (2.1) [High]	4.9 (2.3) [High]	5.1 (2.8) [Very Low]
Between inheritance (combined CNV/SNV)		*t*		-12.48	-10.11	-10.961	-6.101	-6.164	0.312
		*p*-value		< *0.001*	< *0.001*	< *0.001*	< *0.001*	< *0.001*	0.755
**Inheritance of SNV**		***De novo* SNV**	481	17.7 (6.1) [High]	3.7 (2.7) [Close to Average]	2.5 (1.9) [Close to Average]	7.4 (2.2) [Slightly Raised]	4.1 (2.2) [High]	4.7 (2.9) [Very Low]
		**Familial SNV**	84	19.8 (5.1) [High]	4.7 (2.5) [Slightly Raised]	3.1 (1.9) [Slightly Raised]	7.8 (1.8) [Slightly Raised]	4.1 (2.0) [High]	5.4 (2.7) [Very Low]
Between inheritance of SNV		*t*		-3.396	-3.316	-2.829	-1.834	-0.293	-2.099
		*p*-value		< *0.001*	< *0.001*	*0.006*	0.069	0.77	*0.038*
**Inheritance of CNV**		***De novo* CNV**	735	19.2 (6.6) [High]	4.3 (2.8) [Slightly Raised]	3.1 (2.3) [Slightly Raised]	7.5 (2.3) [Slightly Raised]	4.3 (2.3) [High]	5.4 (2.8) [Very Low]
		**Familial CNV**	607	22.8 (6.5) [Very High]	5.5 (2.7) [High]	4.2 (2.5) [High]	8.1 (2.1) [High]	5.0 (2.3) [Very High]	5.1 (2.8) [Very Low]
Between inheritance of CNV		*t*		-9.989	-7.712	-8.515	-5.295	-5.391	2.329
		*p*-value		< *0.001*	< *0.001*	< *0.001*	< *0.001*	< *0.001*	*0.01*

*SDQ* Strengths and Difficulties Questionnaire, *SNV* single nucleotide variant, *CNV* copy number variant, *De novo* non-inherited variant, *Familial* inherited variant.

### Genetic factors and primary special education need (SEN) type

Supplementary Table S1 presents the total number and proportion of participants of different primary SEN types in each inherited/non-inherited variant type. A higher proportion of participants with SNV had profound multiple learning difficulties and severe learning difficulties. Table 3 shows primary SEN types for cohort participants who received EHCPs, broken down into inherited or *de novo* variant types. Nearly all participants with profound multiple learning difficulties or severe learning difficulties had received EHCPs (97–100%, see Table 3), regardless of their variant type or genetic inheritance. When observing the other primary SEN types, a relatively smaller proportion of pupils with familial CNVs had been granted EHCPs within each SEN category (familial CNV: 16.7–75.3%) compared with other groups (*de novo* SNV: 62.5–100%; familial SNV: 76.9–94.7%; *de novo* CNV: 44.4–91.4%).

**Table 3:** The number and proportion of participants who received EHCP with different primary SEN types in each inherited or non-inherited variant type.

**Primary SEN type**	Number (N_EHCP_/N_TOTAL_ %) of participants who had EHCP in each category			
	*De novo* SNV	Familial SNV	*De novo* CNV	Familial CNV
Profound multiple LD	95 (100%)	< 10 (100%)	67 (97.1%)	16 (100%)
Severe LD	198 (100%)	34 (100%)	194 (99.0%)	98 (97%)
Moderate LD	50 (87.7%)	10 (76.9%)	102 (81.0%)	78 (59.5%)
Specific LD	27 (93.1%)	< 10 (85.7%)	33 (62.3%)	33 (50%)
Speech Language Communication Needs	56 (98.2%)	10 (83.3%)	118 (72.8%)	82 (60.3%)
Social, Emotional and Mental Health*	< 10 (100%)	0	10 (45.5%)	15 (37.5%)
Autism Spectrum Disorder	49 (96.1%)	18 (94.7%)	105 (91.4%)	126 (75.4%)
Multiple Sensory Impairment + Hearing/Visual Impairment	< 10 (100%)	0	< 10 (44.4%)	< 10 (16.7%)
Physical Disability	11 (73.3%)	0	16 (64%)	< 10 (41.7%)
Other primary SEN types	< 10 (62.5%)	0	13 (56.5%)	< 10 (27.3%)
Total	501 (96.3%)	84 (89.4%)	662 (81.3%)	460 (66%)

*LD* learning difficulty, *SEN* special education needs, *SNV* single nucleotide variant, *CNV* copy number variant.

*This type was called “Behavioural Emotional Social Difficulty” before 2014–2015. N_EHCP_ = number of participants who had EHCP in each category; N_TOTAL_ = total number of participants in each category (see Supplementary Table S2).

### EHCP obtainment and waiting time

By the time of ascertainment of this study, 2131 pupils (77.8%) had received an EHCP. The mean age at which participants were granted an EHCP was 7.1 (2.6) years (95% CI = 7.0–7.2), with no significant sex differences, *t*(2130) = -0.487, *p* = 0.626, *d* = 2.645. The average waiting time for an EHCP was 1.7 (2.2) years (95% CI = 1.6–1.8) with no sex difference, *t*(2130) = 0.128, *p* = 0.898, *d* = 0.003.

### Genetic factors and EHCP obtainment

**Fig. 1:**
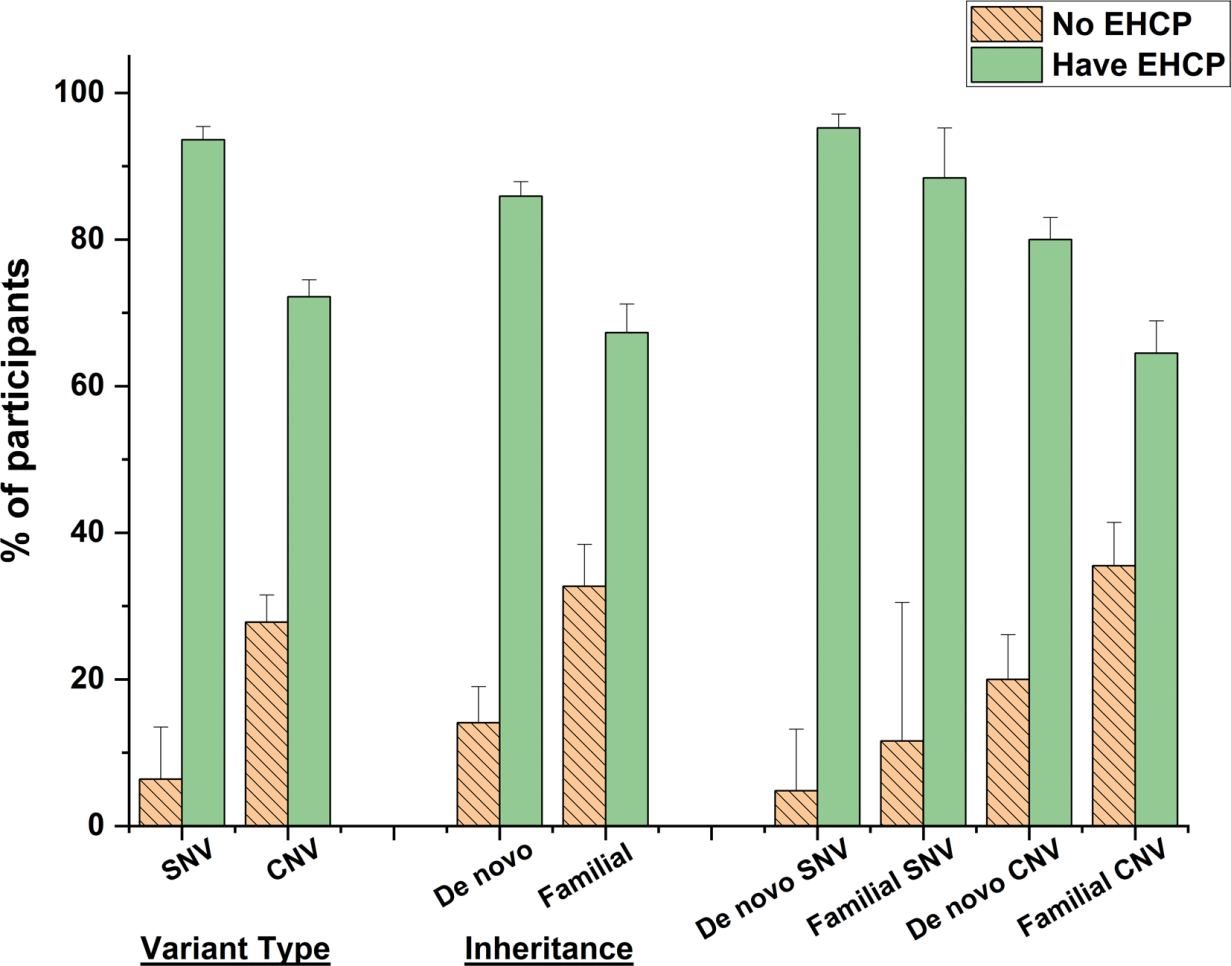
Comparisons of EHCP obtainment of the cohort participants with different variant types and genetic inheritance of the variants, with 95% confidence interval error bars. *EHCP* education health and care plan, *SNV* single nucleotide variant, *CNV* copy number variant, *De novo* non-inherited variant, *Familial* inherited variant.

At the time of recruitment into our study, a higher proportion (93.6%) of participants diagnosed with SNV had been granted an EHCP compared to those with CNV (72.2%), *χ*2 = 140.565, *p* < 0.001, PHI = 0.227 (see Fig. 1). Overall, those with *de novo* variants (85.9%) were more likely to have been granted an EHCP compared to those with familial variants (67.2%), *χ*2 = 104.983, *p* < 0.001, PHI = 0.220. As shown in Table 1, the mean GAC scores for those with *de novo* or familial conditions were very similar, so this did not reflect substantial differences in the severity of their developmental delay. It is notable that those with a familial CNV were far less likely to have been granted an EHCP (64.5%) than those with a *de novo* SNV (95.2%), *χ*2 = 179.102, *p* < 0.001, PHI = 0.288. This is surprising as data in Tables 1 and 2 demonstrate that there were minor differences in the two groups in terms of GAC scores, and those with an inherited CNV tended to have more severe behaviour problems.

### Genetic factors and EHCP waiting times

The EHCP waiting time for participants with a *de novo* or familial CNV [2.0 (2.3) years] was twice as long as those with a SNV [1.0 (1.7) years], *t*(2130) = −10.978, *p* < 0.001, *d* = 2.14. In general, participants with familial CNV or SNV had to wait a significantly longer time to receive an EHCP than those with *de novo* variants [2.2 (2.5) years compared with 1.3 (1.8) years], *t*(1706) = −8.101, *p* < 0.001, *d* = 2.056.

Participants with a familial CNV who received an EHCP waited a mean of 2.4 (2.5) years from the age at which they were recognised as having SEN. Those with a *de novo* CNV waited a mean of 1.6 (2.0) years. Children with an inherited SNV waited a mean of 1.3 (2.0) years and those with a *de novo* SNV waited a mean of 0.9 (1.5) years. The EHCP waiting time were significantly different among these four groups *F*(3, 1703) = 45.917, *p* < 0.001.

### Genetic inheritance, socioeconomic status (SES) and EHCP obtainment

We examined whether genetic inheritance was associated with family socioeconomic status. Pupils with familial variants were more likely to live in lower index of multiple deprivation (IMD) deciles, i.e. more deprived areas (*X*^2^(9) = 125.423, *p* < 0.001, see Supplementary Table S2), and proportionately more of them were eligible for Free School Meals (FSM) (*X*^2^(9) = 334.827, *p* < 0.001). Overall, twice as many children (49%) with familial variants were eligible for FSM compared to those with *de novo* variants (25%), *X*^2^ = 126.602, *p* < 0.001, PHI = 0.242 (see Supplementary Table S2). The proportion of those with *de novo* variants was very similar to the national figure (25.7% pupils with FSM eligibility in England in 2025)^30^.

**Fig. 2:**
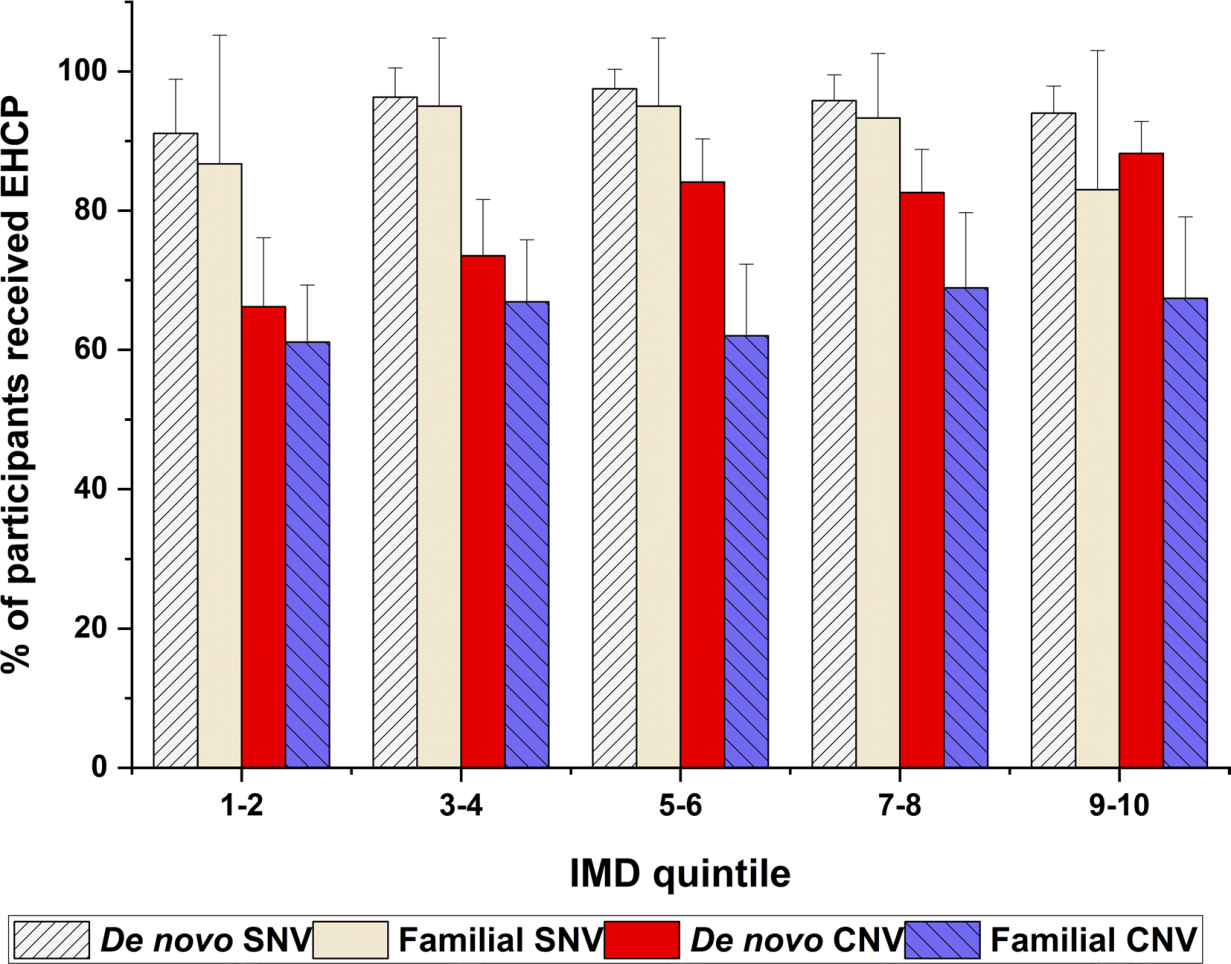
Distribution of the participants with different variant types who were granted with EHCP in the index of multiple deprivation (IMD) quintile areas, with 95% confidence interval error bars. IMD quintiles 1–2 = more deprived areas; IMD quintiles 9–10 = least deprived areas. EHCP = education health and care plan; *SNV* single nucleotide variant, *CNV* copy number variant, *De novo* non-inherited variant, *Familial* inherited variant.

Participants with *de novo* CNV living in more socioeconomically deprived IMD quintiles were less likely to have been granted an EHCP than those with a *de novo* SNV, *X*^2^(4) = 23.69, *p* = 0.017 (66.2–88.2%, see Fig. 2 and Supplementary Table S3). Those with familial CNV had the lowest proportions of participants (61.1%-68.9%) to have been granted an EHCP than those with the other variants no matter in which areas they lived. Overall, a higher proportion of participants with *de novo* or familial SNV (83%-97.5%) had been granted an EHCP whatever IMD quintile they resided (see details in Supplementary Table S3).

**Fig. 3:**
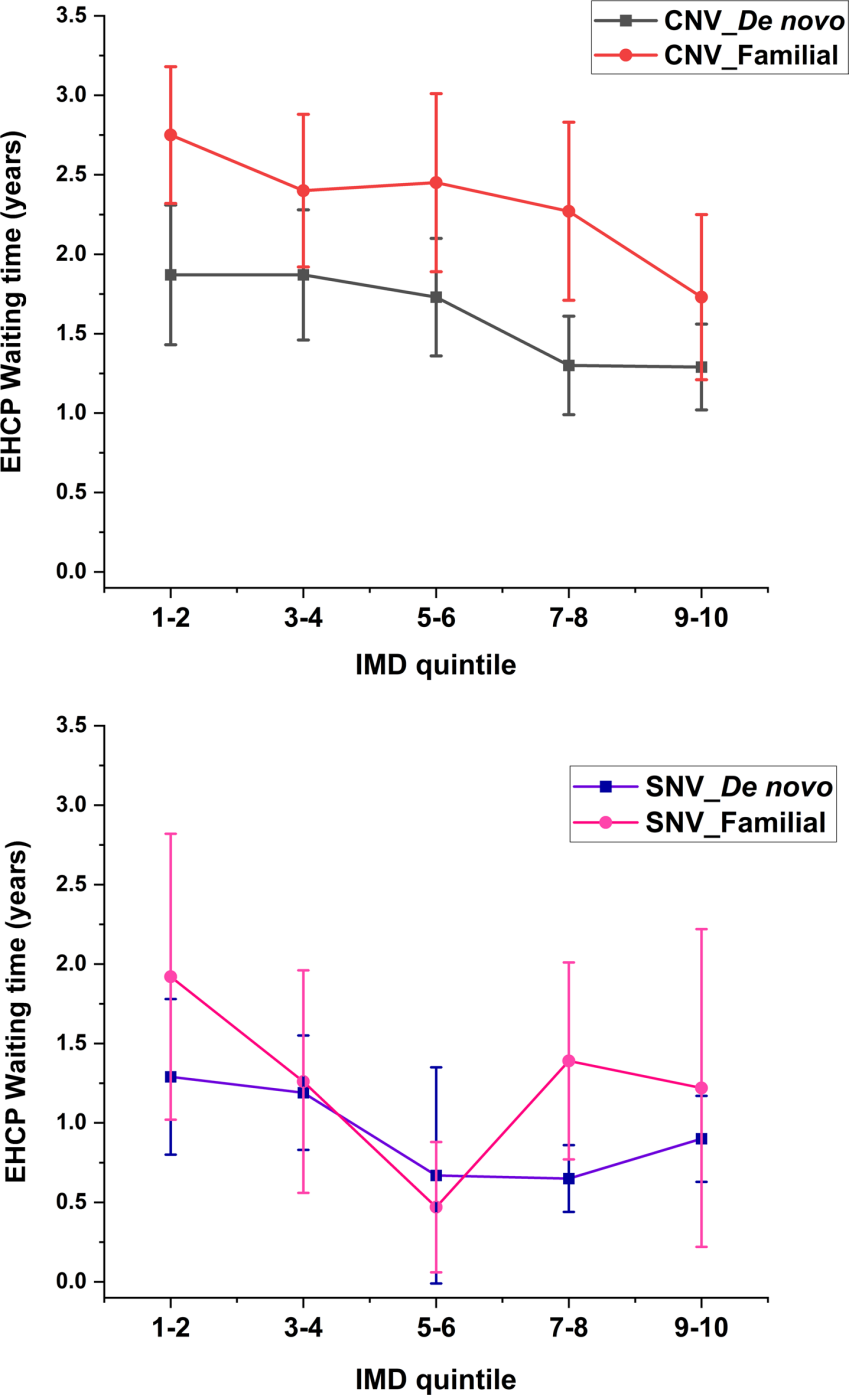
Comparisons of the EHCP waiting time for participants with different genetic inheritance (*De novo*/Familial) of variant types (CNV/SNV). Means and 95% confidence error bars are presented. *EHCP* education health and care plan, *SNV* single nucleotide variant, *CNV* copy number variant, *De novo* non-inherited variant, *Familial* inherited variant.

General linear model analysis showed that participants with familial CNV waited significantly longer for an EHCP than those with *de novo* CNV at the IMD quintiles, *F*(1, 1121) = 50.742, *p* = 0.002 (see Fig. 3 top panel), whereas no significant differences between participants with familial SNV and *de novo* SNV was found, *F*(1, 584) = 2.559, *p* = 0.174 (see Fig. 3 bottom panel).

### Parental education level and EHCP obtainment

**Fig. 4:**
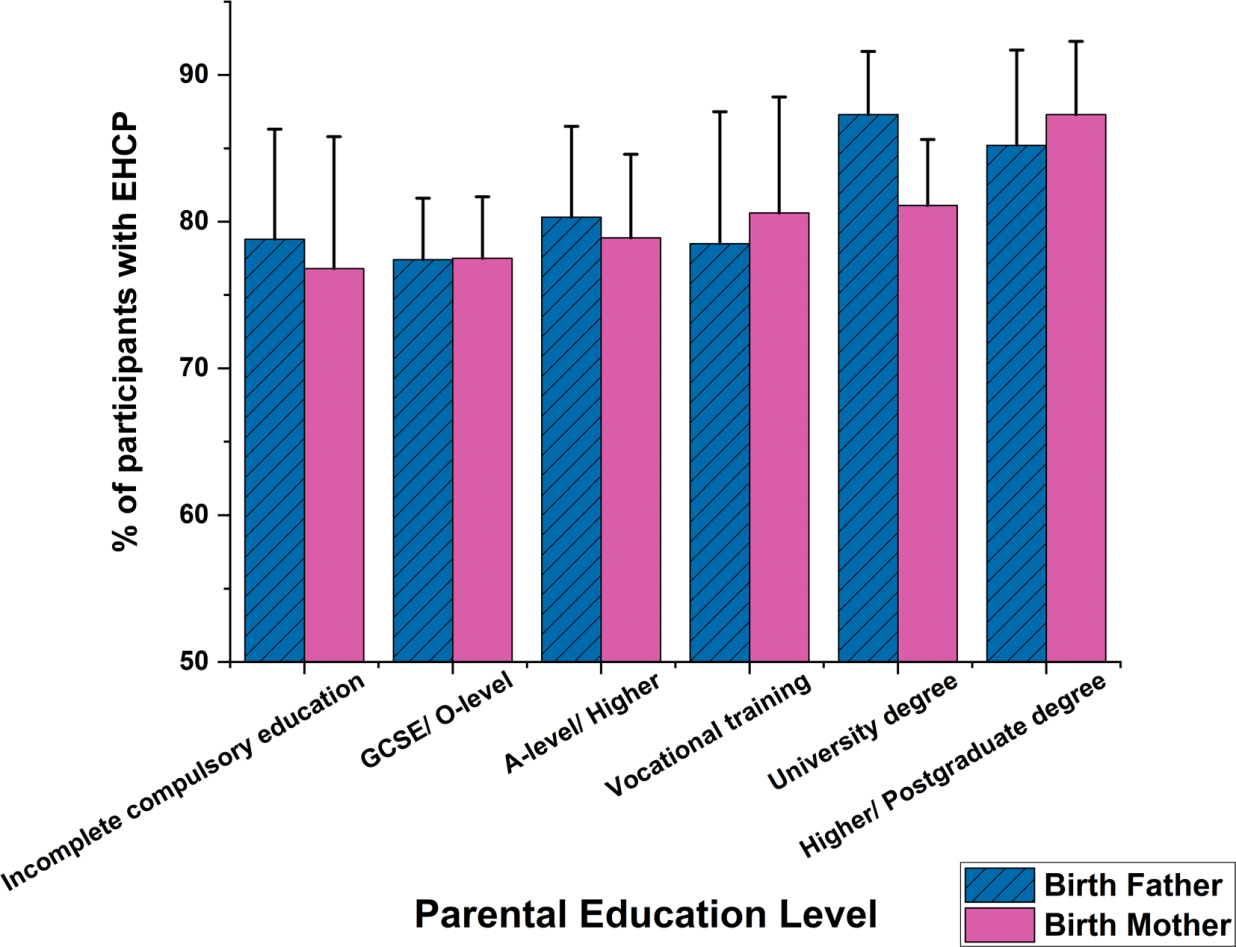
Distribution of the cohort proportion with EHCP against different parental education levels, with 95% confidence interval error bars. *EHCP* education heath care plan, *GCSE* General Certificate of Secondary Education Qualification, *O-level* Ordinary Level Qualification, *A-level* Advanced Level Qualifications.

**Fig. 5:**
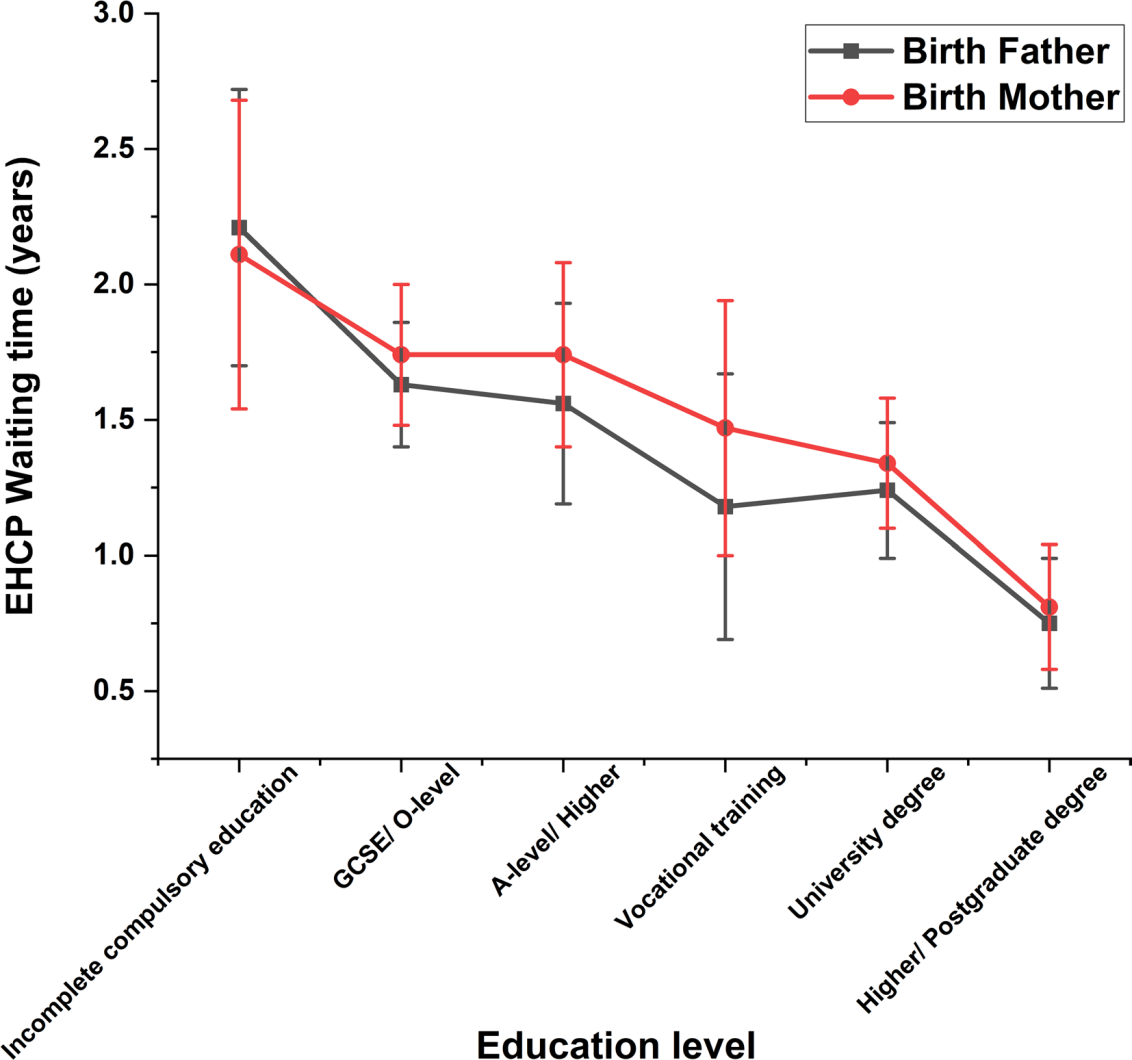
Figure 5 displays the EHCP waiting time of participants against their parental education levels. Mean and 95% confidence interval error bars are presented. *EHCP* education heath care plan, *GCSE* General Certificate of Secondary Education Qualification, *O-level* Ordinary Level Qualification, *A-level* Advanced Level Qualifications.

We investigated whether the education level of the birth fathers or mothers affected the outcome of the EHCP obtainment and waiting time. Pupils whose birth fathers had received higher education (post-school college or university attendance) were more likely to have been granted an EHCP, *X*^2^(5) = 18.249, *p* = 0.006, N = 1242 (see Fig. 4), than those whose fathers had lower educational achievements. No equivalent differences were found for birth mothers, *X*^2^(5) = 8.713, *p* = 0.19, N = 1242. Waiting times for the provision of an EHCP were shorter for the children whose parents had received higher education (1.3–1.5 years), *F*(5, 885) = 7.007, *p* < 0.001 for birth father; *F*(5, 988) = 6.317, *p* < 0.001 for birth mother (see Fig. 5).

The proportion of parents who had achieved a university education or higher degree was substantially lower if the child’s genetic anomaly was inherited (from birth fathers: *X*^2^(5) = 87.683, *p* < 0.001, N = 1024; from birth mothers: *X*^2^(5) = 69.366, *p* < 0.001, N = 1112, see Supplementary Figure S1). Parental education level did not significantly influence the proportion of participants with either *de novo* or familial variants whose child was granted an EHCP. Nevertheless, participants with *de novo* variants and whose parents had experienced higher education obtained an EHCP more quickly (for birth fathers: *F* = 3.61, *p* = 0.003; for birth mothers: *F* = 5.744, *p* < 0.001, see Supplementary Figure S2). In contrast, overall the EHCP waiting time for children with inherited variants was not influenced by their parents’ education level (*p*-values > 0.213), but in general they waited longer than those with *de novo* variants.

### Regression analysis and the predictors

A hierarchical logistic regression analysis showed that variant type, genetic inheritance and socioeconomic deprivation (IMD decile) were significant predictors of EHCP obtainment. These associations remained significant in fully adjusted models that accounted for the child’s developmental level, parental education and child characteristics (see Model 9 in Table 4). In the fully adjusted model, the odds of a child with an SNV obtaining an EHCP were almost four times higher than the odds of a child with a CNV being granted an EHCP (OR = 3.831, 95% CI = 2.12–6.94, *p* < 0.001). The odds of those with a *de novo* variant obtaining an EHCP were nearly two times higher than those with a familial variant (OR = 1.695, 95% CI = 1.08–2.66, *p* = 0.022). The cohort children from less deprived areas (higher IMD decile) were significantly more likely to obtain an EHCP. Each step up the deprivation scale corresponds to roughly a 14% increase in the odds of receiving one (OR = 1.145, 95% CI = 1.06–1.23, *p* < 0.001). Children with higher GAC standard and SDQ total difficulty scores were significantly less likely to receive an EHCP. Each extra point on each score is linked to about 8 and 5% reduction respectively in the odds of obtaining an EHCP.

**Table 4:** Logistic and linear regression models to estimate the effects of predictors on education health and care plan (EHCP) obtainment and waiting time respectively for the cohort pupils.

Analytical model Outcome	Logistic regression EHCP obtainment			Linear regression EHCP waiting time		
Independent variable	B (SE)	OR (95% CI)	*p*-value	B (SE)	β	*p*-value
**Model 1** [N = 2738]	[R^2^ = 9.2%]			[R^2^ = 5.1%]		
**Variant type** (CNV as reference)						
SNV	1.728 (0.16)	5.629 (4.11–7.71)	< *0.001*	-0.214 (0.02)	-0.227	< *0.001*
**Model 2** [N = 2162]	[R^2^ = 7.2%]			[R^2^ = 7.2%]		
**Genetic inheritance** (familial as reference)						
*De novo*	1.083 (0.11)	2.96 (2.39–3.65)	< *0.001*	-0.672 (0.01)	-0.149	< *0.001*
**Model 3** [N = 2738]	[R^2^ = 3.5%]			[R^2^ = 2.3%]		
**Index of multiple deprivation decile**^a^	0.127 (0.02)	1.136 (1.10–1.17)	< *0.001*	-0.021 (0.003)	-0.152	< *0.001*
**Model 4** [N = 1091]	[R^2^ = 1.4%]			[R^2^ = 1.0%]		
**Father education level**	0.132 (0.06)	1.142 (1.02–1.28)	*0.025*	-0.02 (0.01)	-0.082	*0.026*
**Mother education level**	0.026 (0.06)	1.026 (0.91–1.15)	0.196	-0.004 (0.01)	-0.016	0.656
**Model 5** [N = 2162]	[R^2^ = 13.6%]			[R^2^ = 8.1%]		
**Variant type** (CNV as reference)						
SNV	1.561 (0.19)	4.762 (3.32–6.84)	< *0.001*	-0.171 (0.02)	-0.190	< *0.001*
**Genetic inheritance** (familial as reference)						
*De novo*	0.80 (0.77)	2.227 (1.79–2.77)	< *0.001*	-0.139 (0.02)	-0.165	< *0.001*
**Model 6** [N = 2162]	[R^2^ = 14.8%]			[R^2^ = 8.8%]		
**Variant type** (CNV as reference)						
SNV	1.515 (0.19)	4.550 (3.16–6.54)	< *0.001*	-0.163 (0.02)	-0.181	< *0.001*
**Genetic inheritance** (familial as reference)						
*De novo*	0.702 (0.12)	2.018 (1.61–2.53)	< *0.001*	-0.123 (0.02)	-0.146	< *0.001*
**Index of multiple deprivation decile**^a^	0.083 (0.02)	1.086 (1.05–1.13)	< *0.001*	-0.013 (0.003)	-0.093	< *0.001*
**Model 7** [N = 1018]	[R^2^ = 14.3%]			[R^2^ = 8.1%]		
**Variant type** (CNV as reference)						
SNV	1.343 (0.25)	3.83 (2.37–6.20)	< *0.001*	-0.143 (0.03)	-0.176	< *0.001*
**Genetic inheritance** (familial as reference)						
*De novo*	0.66 (0.18)	1.926 (1.34–2.76)	< *0.001*	-0.112 (0.03)	-0.132	< *0.001*
**Index of multiple deprivation decile**^a^	0.115 (0.03)	1.122 (1.06–1.19)	< *0.001*	-0.017 (0.004)	-0.123	< *0.001*
**Father education level**	0.051 (0.06)	1.053 (0.93–1.19)	0.425	-0.001 (0.01)	-0.004	0.915
**Mother education level**	-0.040 (0.06)	0.961 (0.85–1.09)	0.531	-0.008 (0.01)	-0.034	0.350
**Model 8 **[N = 901]	[R^2^ = 36.7%]			[R^2^ = 22.3%]		
**Variant type** (CNV as reference)						
SNV	1.134 (0.28)	3.107 (1.78–5.42)	< *0.001*	-0.081 (0.03)	-0.101	*0.001*
**Genetic inheritance** (familial as reference)						
*De novo*	0.552 (0.23)	1.737 (1.12–2.70)	*0.014*	-0.087 (0.03)	-0.102	*0.002*
**Index of multiple deprivation decile**^a^	0.136 (0.04)	1.146 (1.07–1.23)	< *0.001*	-0.017 (0.004)	-0.125	< *0.001*
**Father education level**	0.012 (0.08)	1.012 (0.97–1.18)	0.882	-0.001 (0.01)	0.000	0.991
**Mother education level**	−0.025 (0.08)	0.976 (0.84–1.14)	0.749	-0.003 (0.01)	-0.012	0.734
**GAC standard score**	-0.92 (0.01)	0.912 (0.90–0.93)	< *0.001*	-0.011 (0.001)	-0.399	< *0.001*
**SDQ total difficulties score**	−0.061 (0.02)	0.941 (0.91–0.97)	< *0.001*	-0.006 (0.002)	-0.089	*0.007*
**Model 9** [N = 871]	[R^2^ = 37.0%]			[R^2^ = 22.4%]		
**Variant type** (CNV as reference)						
SNV	1.343 (0.30)	3.831 (2.12–6.94)	< *0.001*	-0.109 (0.03)	-0.133	< *0.001*
**Genetic inheritance** (familial as reference)						
*De novo*	0.528 (0.23)	1.695 (1.08–2.66)	*0.022*	-0.082 (0.03)	-0.096	*0.004*
**Index of multiple deprivation decile**^a^	0.135 (0.04)	1.145 (1.06–1.23)	< *0.001*	-0.016 (0.004)	-0.121	< *0.001*
**Father education level**	0.003 (0.08)	1.003 (0.86–1.17)	0.969	-0.001 (0.01)	-0.003	0.945
**Mother education level**	-0.035 (0.08)	0.965 (0.83–1.13)	0.652	-0.004 (0.01)	-0.018	0.622
**GAC standard score**	-0.091 (0.01)	0.913 (0.90–0.93)	< *0.001*	-0.011 (0.001)	-0.400	< *0.001*
**SDQ total difficulties score**	-0.057 (0.02)	0.945 (0.91–0.98)	*0.001*	-0.005 (0.002)	-0.083	*0.013*
**Sex**	0.125 (0.21)	0.882 (0.59–1.33)	0.548	-0.007 (0.02)	-0.008	0.779
**Age at diagnosis**	-0.052 (0.03)	0.944 (0.90–1.00)	0.065	-0.006 (0.003)	-0.064	0.051

*CNV* copy number variant; *SNV* single nucleotide variant, *familial* variant inherited from parents, *De novo* non-inherited variant, *GAC* General Adaptive Composite, *SDQ* Strength and Difficulties Questionnaire. *B* Beta, *SE* standard error, *OR* odd ratio, *CI* confidence interval, *β* standardised beta, *R*^2^ = adjusted R square.

^a^The regression results of the variable, Index of multiple deprivation decile, can be found in Lee et al.(2024) and Lee et al. (2025)^16,23^.

The hierarchical linear regression models showed there was also a significant association between genetic factors (variant type and inheritance), socioeconomic deprivation and EHCP waiting time. These associations remained significant in fully adjusted models. Children with a SNV obtained EHCPs sooner than those with a CNV [B(SE) = −0.109 (0.03), *p* < 0.001, see Model 9 in Table 4]. Children with *de novo* variants had slightly shorter EHCP waiting times than those with familial variants [B(SE = -0.082 (0.03), *p* < 0.022]. Participants living in less deprived areas tended to have shorter EHCP waiting times [B(SE) = −0.016 (0.004), p < 0.001]. Higher GAC standard score and SDQ total difficulty scores were associated with shorter EHCP waiting times, however, the per-point effects were very small [B(SE) = −0.011 (0.001), *p* < 0.001 and B(SE) = −0.005 (0.002), *p* = 0.013 respectively].

## Discussion

This is the first report to reveal the impact of genetic inheritance on EHCP obtainment for children and young people with intellectual developmental disabilities of genetic origin. We provide evidence that children whose genetic anomaly is an inherited CNV are significantly less likely to receive timely EHCP support than children with *de novo* variants, irrespective of their level of adaptive functioning or emotional and behavioural adjustment. This differentiation does not apply to those with an SNV. We also show that, in general, those whose genetic disorder is inherited tend to live in areas of relatively greater socioeconomic deprivation, and their parents are less likely to have completed higher education.

Children from less advantaged socioeconomic backgrounds waited longer to receive an EHCP, irrespective of their degree of overall functioning or behavioural and emotional adjustment. It is well known that socioeconomically disadvantaged children are less likely to be supported in their education^16,23,31–33^. However, this study reveals that children with IDD of familial genetic origin are doubly disadvantaged by their inherited genetic risk of learning disabilities and socioeconomic disadvantage.

Most pupils in this study had extremely low general adaptive daily life skills. Participants with familial variants had more emotional and behavioural maladjustment, and therefore an enhanced need for support. Surprisingly, the regression results showed that those with lower daily life skills and higher emotional and behavioural difficulties were less likely to get an EHCP. Placement in special schools for children with IDD requires an EHCP, so children without an EHCP all attended mainstream establishments (a few with specialised units). Pupils with SEN are eight to nine times more likely to be permanently excluded from mainstream schools than their typical peers^26,34^, and they are some of the most socially excluded and bullied pupils in the school system^35,36^. Research studies show that both SNVs and CNVs are strongly associated with neurodevelopmental and psychiatric outcomes^17,37–39^ and can have variable physical phenotypes and multiple medical complications beyond neurodevelopment, including syndromic presentations, cardiac, endocrine or other problems^37,40^. In our cohort, those with an SNV (whether inherited or *de novo*) tended to have more severe learning disabilities and were more likely to receive an EHCP and placement outside mainstream education: they were diagnosed at a later age than those with a CNV because the relevant technology had not been available earlier^1^, not because their condition was less visible.

Our results in Table 2 show that almost all the groups with familial variants had greater behavioural and emotional difficulties than those with *de novo* variants. Huang et al. (2024) describe how parental polygenic and rare-variant burdens interact, providing a mechanism by which familial variants combine with parental liability to produce greater behavioural/emotional difficulties in offspring^41^. Research studies have compared children with inherited CNV (such as 16p11.2 deletion) with those that are *de novo*, and with their unaffected family members^42,43^. The inherited individuals often show milder or more variable behavioural and cognitive features than *de novo* probands, but parental carriers frequently have detectable neurodevelopmental or psychiatric traits themselves^42,43^. This suggests that familial traits and inheritance shape behavioural variability.

We discovered a complex association between genetic inheritance and socioeconomic factors. In general, children carrying an inherited variant had parents with a lower education level and they lived in less advantaged areas. Familial factors are therefore likely to have indirectly impacted those children’s general adaptive functioning and emotional and behavioural adjustment, in addition to the direct influence of their genetic disorder^31,44,45^. Previous studies show that inherited CNV carriers have greater neuropsychiatric and behavioural risk than *de novo* carriers, and familial carriers tended to live in more socioeconomically deprived areas suggesting both inheritance and deprivation contribute to neuropsychiatric risk^17^.

In our cohort, those with familial variants, who tended to have received lower education level, were less likely to obtain an EHCP. Research has found that lower levels of education and lower family income can negatively impact parental self-efficacy and confidence to navigate SEND systems^46^. To advocate for their child’s educational support, families need to be knowledgeable about SEND to navigate the assessment and support system and typically invest substantial time and energy in educating themselves about the process^47,48^. Parents with higher educational attainments are more able to advocate for their child and therefore are more likely to have the resources to appeal to their local education authority^49^. Different parental advocacy styles have been recognised when negotiating with school services for their SEN children^48^. Many (30%) parents feel overwhelmed, burned out or frustrated with the lack of support and generally dissatisfied with the outcomes of the educational planning process^48^. Professional support for parental advocacy has been recommended, which can target development of skills and strategies that have worked for successful negotiators^48^, suggesting an extra need from the professionals for supporting those disadvantaged parents. Our results echo the previous findings indicating the importance of genetic nurture effects which were largely explained by observed parental education and socioeconomic status, pointing to their role in environmental pathways shaping child educational outcomes^50,51^.

There are some limitations in this study. We had a relatively smaller proportion of children with an SNV (26%) compared to those with a CNV in this cohort, due to the paucity of exome sequencing services within the NHS at the time of recruitment. In general, families with a child who was identified with an SNV tended to come from more advantaged socioeconomic circumstances, reflecting the limited access to exome sequencing during the period of data collection. In contrast, CNV screening was more widely available. These disparities in access to genomic sequencing persist, predominantly among under-represented and socioeconomically disadvantaged groups^52^. This reflects another area of inequity in our cohort, highlighting a need for further research in this area. In recent years, there has been a major advance in the provision of whole genome sequence testing. Consequently, there will be more children being identified with pathogenic SNVs for further research. Although we adjusted for the child’s developmental level, parental education and child characteristics, we did not include other factors of potential relevance such as physical health complexity, therefore residual and/or unmeasured confounding remains possible. Potentially, this study could be replicated in the future with a higher proportion of SNVs and control for multiple medical complications, which may influence the likelihood and speed of obtaining an EHCP.

Another limitation is incomplete data about genetic inheritance, because both parents were not necessarily available for testing at Regional Genetics Centres. The cohort as a whole was representative of the general population of the UK in terms of socioeconomic status, but whether the child had exome sequencing was biased toward those with higher levels of parental education. Given the limitations of the NPD data provided, the variable ‘EHCP waiting time’ is limited to whole years, as it was calculated as the difference between the year the pupil was first identified as having SEN and the year in which they were granted an EHCP. No exact dates or months are provided by the NPD datasets. Another limitation relates to the necessity of selecting a single primary special educational need type to represent a single pupil. We observed that, in practice, pupils were classified within multiple primary special education need types during their education in different academic years. Parental phenotypic information was limited, and future studies could include more detailed phenotypes such as parental IQ. We cannot generalise to other conditions that are common reasons for EHCP requests, such as ADHD.

## Conclusions

This study was the first to present systematically an observation of genetic, environmental and familial influences on whether children with IDD are granted an EHCP, and on the waiting time from identification of special educational needs and the final decision. We found significant influences on these variables. Those influences had less to do with the severity of the children’s intellectual disability, medical complexity or behavioural problems than whether or not their condition had been inherited, and the socioeconomic and educational history of their families of origin. Parents with genetic abnormalities are likely to have learning challenges themselves and are therefore less likely to have the skills and ability to fight for an EHCP for their children. They are also more likely to be socially deprived which doubly disadvantages them. These results highlight the importance of socioeconomical inequalities in restricting access to appropriate educational services for children and young people with IDD. Our findings indicate the need for advocates to support vulnerable families who deserve to gain an EHCP for their SEN children.

## Methods

### Study participants

Participants were recruited from the Intellectual Disability and Mental Health: Assessing the Genomic Impact on Neurodevelopment (IMAGINE-ID) study^17^. All were over 4 years of age at enrolment, with developmental delay or an intellectual disability diagnosis identified by a clinical care team. Participation required a molecular genetic diagnosis from an accredited diagnostic laboratory^53^. Pathogenic variants were classified according to the American College of Medical Genetics and Genomics guidelines and only those participants with pathogenic or likely pathogenic variants were included^54^. Recruitment to the study was by referral from 23 UK regional genetics centres (76%) and self-referrals or patient support groups (24%).

The present study focused on a subset of 2738 individuals between 6–28 years of age [mean (standard deviation) = 13.8 (4.3); 56% male; see Table 5]^16^. Genetic information included details of variant type and of inheritance (when available). Educational histories were provided by the National Pupil Database (NPD) for England (Scotland, Wales and Northern Ireland have other record systems). This study was approved by the London Square Research Ethics Committee in the UK. Informed consent was obtained from all subjects and/or their legal guardian(s), including for linkage to clinical and educational records. All methods were carried out in accordance with relevant guidelines and regulations.

**Table 5:** Participant demographic information.

Demographic category	Frequency, N (% of total count)	95% CI Cohort proportion
**Sex**		
Male	1535 (56%)	54.2–57.9%
Female	1203 (44%)	42.1–45.8%
Total count	2738	
**Age at recruitment (years)**		
Mean (SD) [95% CI]	13.8 (4.3)	[13.7–14.0]
Median	13.3	
Range	6.4–27.7	
**Variant type**		
SNV	719 (26.3%)	23.1–29.5%
CNV	2019 (73.7%)	71.8–75.6%
**Genetic inheritance**		
*De novo*	1354 (49.5%)	46.8–52.5%
Familial	808(29.5%)	26.4–32.6%
Unknown	576 (21%)	–-
**Variant type x inheritance**		
*SNV – de novo*	*526 (24.3%)*	*20.6–28.0%*
*SNV—Familial*	*95 (4.4%)*	*0.3–8.5%*
*CNV – de novo*	*828 (38.3%)*	*35.0–41.6%*
*CNV – Familial*	*713 (33%)*	*29.5–36.5%*
*Total data count*	*2162*	
**Primary SEN type**		
Profound multiple LD	226 (8.3%)	7.3–9.3%
Severe LD	625 (22.8%)	21.2–24.4%
Moderate LD	437 (16.0%)	14.6–17.4%
Specific LD	208 (7.6%)	6.6–8.6%
Speech Language Communication Needs	474 (17.3%)	15.9–18.7%
Autism Spectrum Disorder	461 (16.8%)	15.4–18.2%
Social, Emotional and Mental Health*	93 (3.4%)	2.7–4.1%
Multiple Sensory Impairment + Hearing/ Visual Impairment	29 (1.1%)	0.7–1.5%
Physical Disabilities	71 (2.6%)	2–3.2%
Other difficulties/disabilities	65 (2.4%)	1.8–3.0%
Missing data	49 (1.8%)	1.3–2.3%
**Index of multiple deprivation decile**		
1 (most deprived)	303 (11.1%)	9.9–12.3%
2	282 (10.3%)	9.2–11.4%
3	269 (9.8%)	8.7–10.9%
4	253 (9.2%)	8.1–10.3%
5	276 (10.1%)	9.0–11.2%
6	263 (9.6%)	8.5–10.7%
7	247 (9.0%)	7.9–10.1%
8	273 (10%)	8.8–11.1%
9	274 (10%)	8.9–11.1%
10 (least deprived)	298 (10.9%)	9.7–12.1%
**Free school meal eligibility**		
No	1774 (64.8%)	63.0–66.6%
Yes	964 (35.2%)	33.4–37.0%

*N* number of cases, *CI* confidence interval, *SD* standard deviation, *SNV* single nucleotide variant, *CNV* copy number variant; *De novo* non-inherited variant, *Familial* inherited variant, *SEN* special educational need, *LD* learning difficulty.

*The type was called “Behavioural Emotional Social Difficulty” before 2014–15.

### Data source and description

All participants had been educated in English mainstream or special educational state-funded schools. Personal data were linked to education histories derived from the NPD which is managed by the UK Department for Education (DfE). The NPD educational dataset was ideal for the investigation of this study as the state schools record information about EHCP provisions. We therefore had access to official records, ensuring accuracy. Educational information was provided between the period from 2006 to 2021 which included primary SEN type, socioeconomic impoverishment (indicated by whether the pupil was eligible for free school meal), the year the pupil was identified as having SEN and the year that pupil was granted an EHCP. A variable “EHCP waiting time” (in years) was estimated by taking the difference in time between the year the pupil’s SEN need for assessment was recognised and the year they received the EHCP. Exact dates were not available in the NPD datasets. NPD records showed that individual pupils were classified as belonging to different ‘primary SEN type’ in consecutive academic years. For the purpose of this analysis, we assigned just one primary SEN type for each pupil, which was the one most frequently recorded in their education history^16^.

Genetic information indicated participants had either a putatively pathogenic copy number variant (CNV) or single nucleotide variant (SNV)^17^. 2019 participants (73.7%, see Table 5) had a CNV and 719 (26.3%) had a SNV. About 80% participants (2162) provided information on the inheritance of the variant, which was categorised as either *de novo* or familial. Familial status was assigned when a variant was inherited from the mother or father of the CYP, whereas *de novo* was assigned when the family had no history of the genetic condition that was present in the CYP. 1354 (49.5%) of CYP participants had *de novo* variant and 808 (29.5%) had familial variant. No information on genetic inheritance was available for the remaining 576 (21%) participants. Few participants had a familial SNV (n = 95, 4.4%).

The primary caregivers of 1242 participants (45%) completed the Adaptive Behaviour Assessment System 3 (ABAS-3) which evaluates daily living skills^27^ and provides information of the parental education level. General Adaptive Composite (GAC) standard scores from ABAS-3 were calculated, which were categorised descriptively into different standard score groups to reveal the individual’s adaptive abilities: scores lower than 70 as ‘Extremely Low’, between 71–80 as ‘Low’, 81–90 as ‘Below Average’, ranged from 91–110 regarded as ‘Average’, 111–120 as ‘Above Average’ and higher than 120 as ‘High’^27^.

2369 primary caregivers (86%) completed the Strengths and Difficulties Questionnaire (SDQ) which is widely used to screen for emotional and behavioural adjustment in population surveys^28^, and is suitable for evaluating emotional and behavioural difficulties in CYP with IDD^55^. The SDQ comprises four problem domain sub-scales: emotional symptoms; conduct problems; hyperactivity, impulsivity and inattention difficulties; and peer relationship problems, plus a measure of prosocial behaviour. Problem scales are combined to yield a total difficulties score^28,56^. SDQ scores have been categorised into four bands based on general population survey data (see Supporting Information in Note S1): 80%of UK children score in a ‘close to average’ range regarded as ‘normal’, 10% score in a ‘slightly raised’ range of problems and are regarded as ‘borderline’. 10% score in the ‘high’ or ‘very high’ range, indicative of problems that have potential clinical significance^29^.

We had permission to use the ABAS-3 and SDQ questionnaires for this study. Parents/carers provided their postcodes which were used to calculate multiple deprivation (IMD) deciles (1 = most deprived, 10 = least deprived) according to the UK Office for National Statistics^57^.

### Statistical analysis

Chi-squared (*X*^2^) tests were used to compare proportions of participants with different categorical variables, such as genetic inheritance, variant type and parental education level. We used parametric statistics to identify the impact of the above independent variables. General linear models were applied to evaluate the associations between genetic inheritance (fixed factor, i.e. *de novo* vs familial) and EHCP waiting time (dependent variable) across different IMD areas (covariate, range from 1 to 10). A series of logistic and linear regression analyses were performed to investigate the influence of variant type, genetic inheritance and socio-economic deprivation on EHCP obtainment and EHCP waiting time respectively. In each set of analyses, we controlled for and both parents’ education level, adaptive behaviour (ABAS GAC), emotional and behavioural adjustment (SDQ), age of genetic diagnosis and sex. A *p*-value < 0.05 was adopted in all the analyses in this study as a cut-off of statistical significance. All data analyses were performed in SPSS version 28 on the Office for National Statistics (ONS) Secure Research Service platform. The ONS stipulate that educational data from the NPD cannot be used to report on variables endorsed by less than ten pupils to protect the privacy of individuals. Therefore, we report figures for certain groups as < 10.

## Supplementary Information

Supplementary Information.

## Data Availability

The data from the Department for Education (DfE), UK that support the findings in this paper are not available for data sharing according to the guidelines of the DfE and the Office for National Statistics (ONS). Other IMAGINE ID research data from this study are available from the authors upon application (see details of the application of data access in https://imagine-id.org/research-data-access/; contact person: Irene Lee, irene.lee@ucl.ac.uk). This work contains statistical data from ONS which is Crown Copyright. The use of the ONS statistical data in this work does not imply the endorsement of the ONS in relation to the interpretation or analysis of the statistical data. This work uses research datasets which may not exactly reproduce National Statistics aggregates.
