# Supplementary material for: The influence of genetic factors on education health and care plan obtainment for pupils with intellectual developmental disabilities
Source: Sci Rep. 2026 Feb 15;16:9181. doi: 10.1038/s41598-026-36227-5 (PMC12996278; doi:10.1038/s41598-026-36227-5)
Supplement: Supplementary file 1 — Supplementary Information. [file 41598_2026_36227_MOESM1_ESM.docx]

**Supplementary Information**

**The influence of genetic factors on Education Health and Care Plan obtainment for pupils with intellectual developmental disabilities**

Irene O Lee^1^, Jeanne Wolstencroft^1^, Harriet Housby^2^, Marianne B M van den Bree^3,4^, Samuel J R A Chawner^3^, Jeremy Hall^3,4^, Michael J Owen^3,4^, IMAGINE ID Consortium^5^ and David H Skuse^1^

^1^ Paediatric Mental Health Sciences Centre, Great Ormond Street Institute of Child Health, University College London, London, UK.

^2^ The Division of Psychology and Language Sciences, Faculty of Brain Sciences, University College London, London, UK.

^3^ Centre for Neuropsychiatric Genetics and Genomics, Division of Psychological Medicine and Clinical Neurosciences, Cardiff University, Cardiff, UK.

^4^ Neuroscience and Mental Health Innovation Institute, Division of Psychological Medicine and Clinical Neurosciences, Cardiff University, Cardiff, UK.

^5^ IMAGINE ID Consortium, GB, UK.

**Table of Contents**

[**IMAGINE ID Consortium GB** 2](#_Toc211422796)

[**Supplementary Note S1.** More information about the SDQ measures. 3](#_Toc211422797)

[**Supplementary Table S1.** The total number and proportion of participants of different primary special education needs (SEN) types in each inherited or non-inherited variant type**.** 5](#_Toc211422798)

[**Supplementary Table S2.** Distribution of the cohort participants with different genetic inheritance of the variants (*de novo* or familial) in the ten index of multiple deprivation (IMD) deciles and that of free school meal (FSM) eligibility. 6](#_Toc211422799)

[**Supplementary Table S3.** Comparisons of participants with different variant groups who were granted with education health and care plan (EHCP) in the index of multiple deprivation (IMD) areas. 7](#_Toc211422800)

[**Supplementary Figure S1.** Comparisons of parental education levels of the cohort participants with different genetic inheritance. 8](#_Toc211422801)

[**Supplementary Figure S2.** Comparisons of parental education levels of the cohort participants with different genetic inheritance to obtain an education health and care plan (EHCP). 9](#_Toc211422802)

[**References** 10](#_Toc211422803)

# **IMAGINE ID Consortium GB**

David Skuse^1,^******

Jeanne Wolstencroft^1^

Irene O Lee^1^

Samuel Chawner^3^

Jeremy Hall^3,4^

Josh Hope-Bell^3^

Michael J Owen^3,4^

Marianne van den Bree^3,4^

F Lucy Raymond^5^

**** Consortium contact:** David H Skuse, Paediatric Mental Health Sciences Centre, Great Ormond Street Institute of Child Health, University College London, London, UK; Email: [d.skuse@ucl.ac.uk](mailto:d.skuse@ucl.ac.uk)

^1^ Paediatric Mental Health Sciences Centre, Great Ormond Street Institute of Child Health, University College London, London, UK.

^3^ Centre for Neuropsychiatric Genetics and Genomics, Division of Psychological Medicine and Clinical Neurosciences, Cardiff University, Cardiff, UK.

^4^ Neuroscience and Mental Health Innovation Institute, Division of Psychological Medicine and Clinical Neurosciences, Cardiff University, Cardiff, UK.

^5^ Department of Medical Genetics, University of Cambridge, UK

Josh Hope-Bell and F Lucy Raymond did not directly contribute to the manuscript but were involved in other areas of the project.

# **Supplementary Note S1.** More information about the SDQ measures.

The Strengths and Difficulties Questionnaire (SDQ)[1] is a brief behavioural screening questionnaire about 2–17 year olds, and is a globally recognised instrument for assessing the mental health status for children and young people [2]. SDQ scoring provides an overview to evaluate children and young people’s emotional and behavioural adjustment in dimensional terms [3, 4].

There are 25 items in the SDQ which comprises of 5 scales in 5 items each that measure: emotional symptoms; conduct problems; hyperactivity, impulsivity, and inattention difficulties; peer relationship problems; and prosocial behaviour. The first four of these scales are combined to make a total difficulties score. Higher scores are indicative of a greater mental health difficulty and scores above 90th percentile indicate a high probability of a diagnosable psychiatric disorder; whereas a lower score on the prosocial behaviour implies greater difficulties [4, 5]. The SDQ has been used as a standardised assessment to evaluate emotional and behavioural difficulties of children and adolescents with intellectual and developmental disabilities [3].

The SDQ scores were categorised into four bands based on a general population UK survey: 80% of UK children score in a “Close to Average” range regarded as ‘normal’, 10% score “Slightly Raised” range as ‘borderline’, and 10% score “High” or “Very High” range regarded as ‘abnormal’ [5, 6].

Categorisation bands for SDQ scores are listed in the following table to identify the correct classification for each score.

For Parent/Carer completed SDQ:

| **Classification for each SDQ subscale score** | **Close to**  **Average**  (80% pop) | **Slightly raised**  **(/lowered)**  (10% pop) | **High**  **(/Low)**  (5% pop | **Very high**  **(/very low)**  (5% pop) |
| --- | --- | --- | --- | --- |
| Emotional problems score | 0-3 | 4 | 5-6 | 7-10 |
| Conduct problems score | 0-2 | 3 | 4-5 | 6-10 |
| Hyperactivity score | 0-5 | 6-7 | 8 | 9-10 |
| Peer problems score | 0-2 | 3 | 4 | 5-10 |
| Prosocial score | 8-10 | 7 | 6 | 0-5 |
| **Total difficulties score** | 0-13 | 14-16 | 17-19 | 20-40 |

Note: pop=population.

For further information regarding the scoring process, please visit: <https://sdqscore.org/>.

**Supplementary Table S1.** The total number and proportion of participants of different primary special education needs (SEN) types in each inherited or non-inherited variant type**.**

|  | **Total number of participants in each category (% of participants in each variant group total)** | | | |
| --- | --- | --- | --- | --- |
| **Primary SEN Types** | ***De novo* SNV** | **Familial SNV** | ***De novo* CNV** | **Familial CNV** |
| **Profound Multiple LD** | 95 (18.3%) | <10 (<11%) | 69 (8.5%) | 16 (2.3%) |
| **Severe LD** | 198 (38.1%) | 34 (36.2%) | 196 (24.1%) | 101 (14.5%) |
| **Moderate LD** | 57 (11.0%) | 13 (13.8%) | 126 (15.5%) | 131 (18.8%) |
| **Specific LD** | 29 (5.6%) | <10 (<11%) | 53 (6.5%) | 66 (9.5%) |
| **Speech Language Communication Needs** | 57 (11.0%) | 12 (12.8%) | 162 (19.9%) | 136 (19.5%) |
| **Social, Emotional and Mental Health *** | <10 (<2%) | 0 (0%) | <25 (<3.1%) | 40 (5.7%) |
| **Autism Spectrum Disorder** | 51 (9.8%) | 19 (20.2%) | 129 (15.8%) | 167 (24.0%) |
| **Multiple Sensory Impairment +Hearing/Visual Impairment** | <10 (<2%) | 0 (0%) | <10 (<1.2%) | <10 (<1.2%) |
| **Physical Disability** | 15 (2.9%) | <10 (<11%) | 25 (3.1%) | <15 (<2%) |
| **Other primary SEN types** | <10 (<2%) | <10 (<11%) | 23 (2.8%) | 22 (3.2%) |
| **Total** | 520 (100%) | 94 (100%) | 814 (100%) | 697 (100%) |

LD=Learning Difficulty; SEN=Special Education Needs; SNV=Single Nucleotide Variant; CNV=Copy Number Variant. *This type was called “Behavioural Emotional Social Difficulty” before 2014-2015. (%) shows the top three primary SEN types in terms of proportions.

**Supplementary Table S2.** Distribution of the cohort participants with different genetic inheritance of the variants (*de novo* or familial) in the ten index of multiple deprivation (IMD) deciles and that of free school meal (FSM) eligibility.

| Genetic inheritance | *De novo* | Familial | *X*^2^ | PHI | *p*-value |
| --- | --- | --- | --- | --- | --- |
| IMD decile |  |  | 125.423 | 0.241 | *<0.001* |
| 1 (most deprived) | 88 (39.3%) | 136 (60.7%) |  |  |  |
| 2 | 101 (50.2%) | 100 (49.8%) |  |  |  |
| 3 | 127 (57.7%) | 93 (42.3%) |  |  |  |
| 4 | 110 (55.8%) | 87 (44.2%) |  |  |  |
| 5 | 132 (61.7%) | 82 (38.3%) |  |  |  |
| 6 | 144 (66.4%) | 73 (33.6%) |  |  |  |
| 7 | 142 (70.0%) | 61 (30.0%) |  |  |  |
| 8 | 148 (67.3%) | 72 (32.7%) |  |  |  |
| 9 | 167 (75.6%) | 54 (24.4%) |  |  |  |
| 10 (least deprived) | 195 (79.6%) | 50 (20.4%) |  |  |  |
|  |  |  |  |  |  |
| FSM eligibility | **No** | **Yes** | ***X*^2^** | **PHI** | ***p*-value** |
| IMD decile |  |  | 334.827 | 0.350 | *<0.001* |
| 1 (most deprived) | 104 (34.3%) | 199 (65.7%) |  |  |  |
| 2 | 137 (48.6%) | 145 (51.4%) |  |  |  |
| 3 | 124 (46.1%) | 145 (53.9%) |  |  |  |
| 4 | 150 (59.3%) | 103 (40.7%) |  |  |  |
| 5 | 187 (67.8%) | 89 (32.2%) |  |  |  |
| 6 | 194 (73.8%) | 69 (26.2%) |  |  |  |
| 7 | 182 (73.7%) | 65 (26.3%) |  |  |  |
| 8 | 218 (79.9%) | 55 (20.1%) |  |  |  |
| 9 | 228 (83.2%) | 46 (16.8%) |  |  |  |
| 10 (least deprived) | 250 (83.9%) | 48 (16.1%) |  |  |  |
|  |  |  |  |  |  |
| Genetic Inheritance |  |  | 126.602 | 0.242 | *<0.001* |
| *De novo* | 1013 (74.8%) | 341 (25.2%) |  |  |  |
| Familial | 413 (51.1%) | 395 (48.9%) |  |  |  |

**Supplementary Table S3.** Comparisons of participants with different variant groups who were granted with education health and care plan (EHCP) in the index of multiple deprivation (IMD) areas. 1-2 = more deprived areas; 9-10 = least deprived areas.

| Number of participants had EHCP/Total number of participants in each category (%) | | | | | |
| --- | --- | --- | --- | --- | --- |
| IMD Quintile | ***De novo* CNV** | **Familial CNV** | ***De novo* SNV** | **Familial SNV** |  |
| 1-2 | 88/133 (66.2%) | 135/221 (61.1%) | 51/56 (91.1%) | 13/15 (86.7%) |  |
| 3-4 | 114/155 (73.5%) | 107/160 (66.9%) | 79/82 (96.3%) | 19/20 (95%) |  |
| 5-6 | 132/157 (84.1%) | 85/137 (62.0%) | 116/119 (97.5%) | 15/18 (83.3%) |  |
| 7-8 | 142/172 (82.6%) | 71/103 (68.9%) | 113/118 (95.8%) | 28/30 (93.3%) |  |
| 9-10 | 186/211 (88.2%) | 62/92 (67.4%) | 142/151 (94%) | <10/12 (<83%) |  |
| Total | 662/828 (80%) | 460/713 (64.5%) | 501/526 (95.2%) | <85/95 (<89.5%) |  |

**Supplementary Figure S1.** Comparisons of parental education levels of the cohort participants with different genetic inheritance.

**
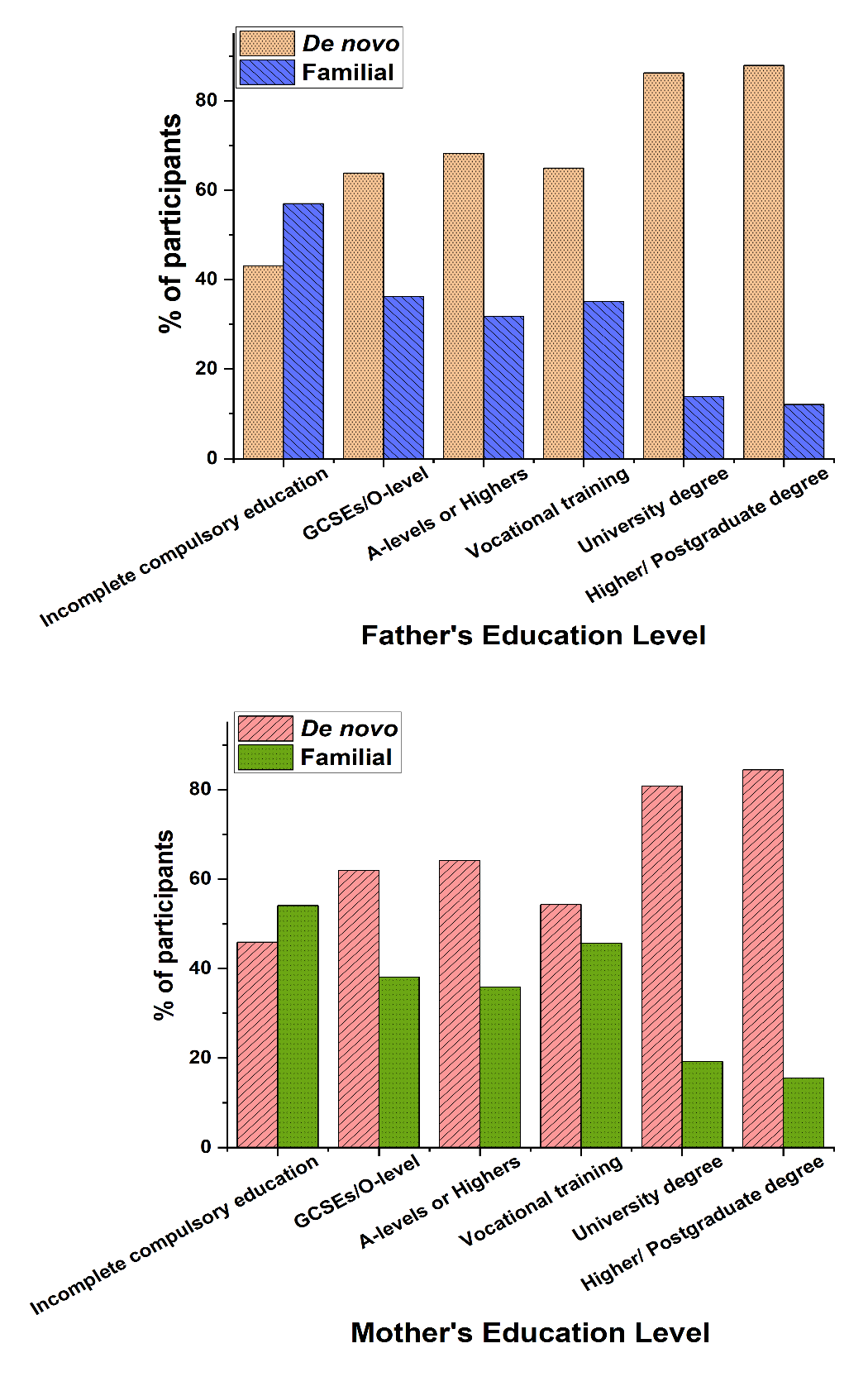
**

Note: GCSE= General Certificate of Secondary Education qualification; O-level= Ordinary Level qualification; A-level=Advanced level qualifications.

**Supplementary Figure S2.** Comparisons of parental education levels of the cohort participants with different genetic inheritance to obtain an education health and care plan (EHCP).

**
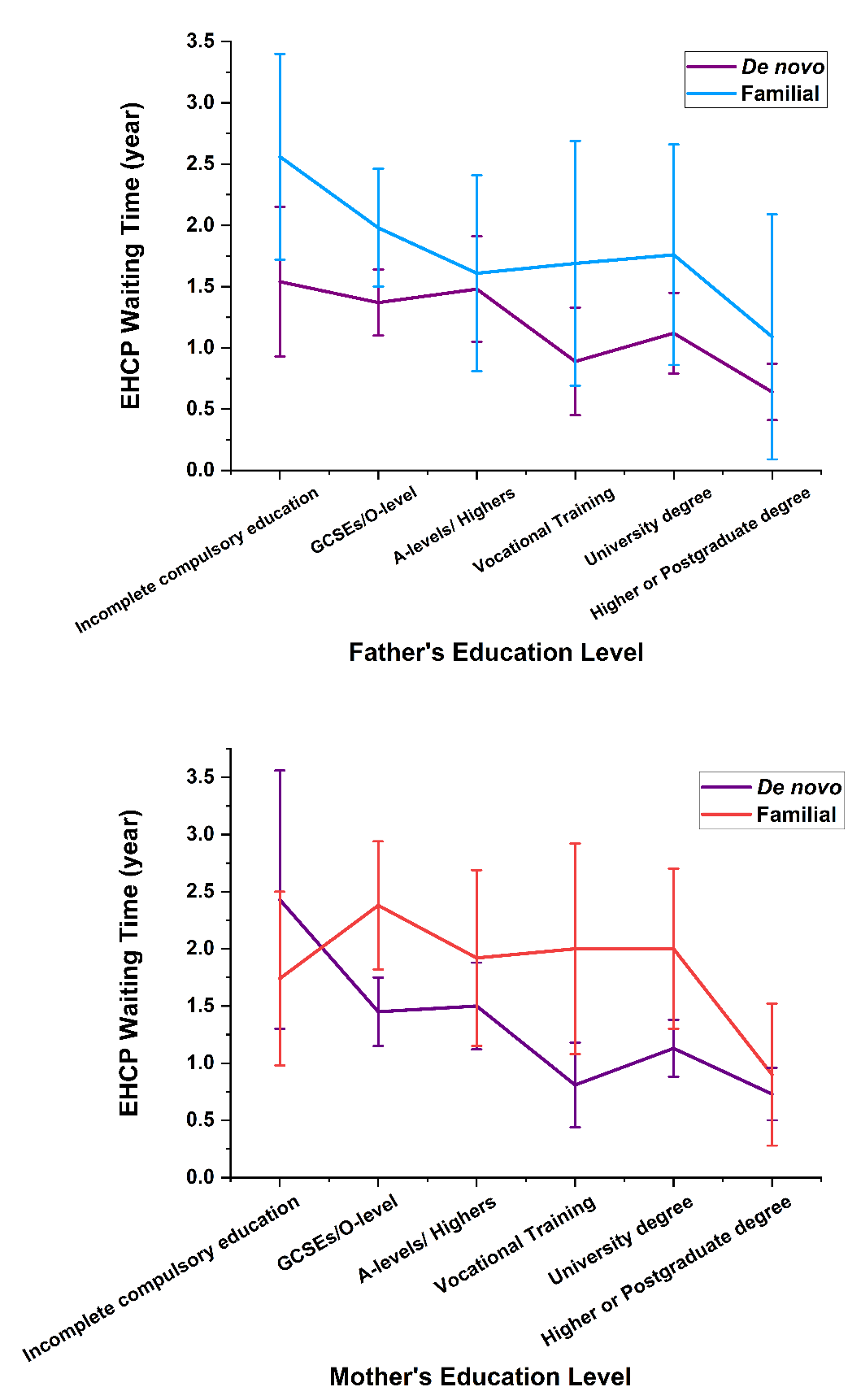
**

Note: GCSE= General Certificate of Secondary Education qualification; O-level= Ordinary Level qualification; A-level=Advanced level qualifications.

**References**

1. Goodman, R. The strengths and difficulties questionnaire: A research note. *J Child Psychol Psychiatry*. **38**, 581–86 (1997). DOI: <https://acamh.onlinelibrary.wiley.com/doi/epdf/10.1111/j.1469-7610.1997.tb01545.x>.

2. Terapia. Scoring strengths and difficulties questionnaire for age 4-17 or 18+ 2020 [Available from: <https://terapia.co.uk/wp-content/uploads/2020/05/SDQ-scoring_Instructions_4-18-years.pdf>.

3. Murray, C. A., Hastings R. P., Totsika V. Clinical utility of the parentreported strengths and difficulties questionnaire as a screen for emotional and behavioural difficulties in children and adolescents with intellectual disability. *Br J Psychiatry*. **218**, 323-25 (2020). DOI: 10.1192/bjp.2020.224.

4. Goodman, A., Goodman R. Strengths and difficulties questionnaire as a dimensional measure of child mental health. *Journal of the American Academy of Child and Adolescent Psychiatry*. **48** (4), 400-3 (2009). DOI: 10.1097/CHI.0b013e3181985068.

5. Goodman, R. Psychometric properties of the strengths and difficulties questionnaire. *J Am Acad Child Adolesc Psychiatry*. **40**, 1337-45 (2001). DOI: 10.1097/00004583-200111000-00015.

6. Youth in Mind. Scoring the strengths and difficulties questionnaire for ages 4–17. In scoring the sdq: Youth in Mind; 2016 [Available from: <https://www.sdqinfo.org/py/sdqinfo/c0.py>.
